# The Efficacy of Renal Replacement Therapy for Rewarming of Patients in Severe Accidental Hypothermia—Systematic Review of the Literature

**DOI:** 10.3390/ijerph18189638

**Published:** 2021-09-13

**Authors:** Konrad Mendrala, Sylweriusz Kosiński, Paweł Podsiadło, Mathieu Pasquier, Peter Paal, Piotr Mazur, Tomasz Darocha

**Affiliations:** 1 Department of Anesthesiology and Intensive Care, Medical University of Silesia, Medykow 14, 40-752 Katowice, Poland; tomekdarocha@wp.pl; 2 Faculty of Health Sciences, Jagiellonian University Medical College, Michalowskiego 12, 31-126 Krakow, Poland; kosa@mp.pl; 3 Institute of Medical Sciences, Jan Kochanowski University, Al. IX Wiekow Kielc 19A, 25-317 Kielce, Poland; p.podsiadlo.01@gmail.com; 4 Emergency Department, Lausanne University Hospital, University of Lausanne, BH 09, CHUV, 1011 Lausanne, Switzerland; mathieu.pasquier@chuv.ch; 5 Department of Anesthesiology and Intensive Care Medicine, St. John of God Hospital, Paracelsus Medical University, Kajetanerplatz 1, 5020 Salzburg, Austria; peter.paal@icloud.com; 6 Department of Cardiovascular Surgery, Mayo Clinic, 200 First St. SW, Rochester, MN 55902, USA; piotr.k.mazur@gmail.com; 7 Institute of Cardiology, Jagiellonian University Medical College, Pradnicka 80, 31-202 Krakow, Poland

**Keywords:** renal replacement therapy, hypothermia, rewarming

## Abstract

Background: Renal replacement therapy (RRT) can be used to rewarm patients in deep hypothermia. However, there is still no clear evidence for the effectiveness of RRT in this group of patients. This systematic review aims to summarize the rewarming rates during RRT in patients in severe hypothermia, below or equal to 32 °C. Methods: This systematic review was registered in the PROSPERO International Prospective Register of Systematic Reviews (identifier CRD42021232821). We searched Embase, Medline, and Cochrane databases using the keywords hypothermia, renal replacement therapy, hemodialysis, hemofiltration, hemodiafiltration, and their abbreviations. The search included only articles in English with no time limit, up until 30 June 2021. Results: From the 795 revised articles, 18 studies including 21 patients, were selected for the final assessment and data extraction. The mean rate of rewarming calculated for all studies combined was 1.9 °C/h (95% CI 1.5–2.3) and did not differ between continuous (2.0 °C/h; 95% CI 0.9–3.0) and intermittent (1.9 °C/h; 95% CI 1.5–2.3) methods (*p* > 0.9). Conclusions: Based on the reviewed literature, it is currently not possible to provide high-quality recommendations for RRT use in specific groups of patients in accidental hypothermia. While RRT appears to be a viable rewarming strategy, the choice of rewarming method should always be determined by the specific clinical circumstances, the available resources, and the current resuscitation guidelines.

## 1. Introduction

The management of the patients suffering from accidental hypothermia, defined as an unintentional drop of the body core temperature below 35 °C, is challenging. The rewarming approach depends mainly on the patient’s core temperature, clinical condition, and environmental setting [1]. In the pre-hospital setting, the highest priority should be given to the early recognition of hypothermia, thermal insulation to prevent further heat loss, and prompt transfer to the hospital [2]. Among the active rewarming methods available in the hospital setting, non-invasive and invasive can be distinguished. The management often combines techniques from different categories. Active external methods include warm blankets or forced warm air, with a rewarming rate of 0.5–4 °C/h. Active internal rewarming includes body cavities lavage with warm fluids (bladder, gastric, thoracic, or peritoneal lavage) with the rewarming rate estimated at 0.5–2 °C/h, application of renal replacement therapy (RRT) or extracorporeal life support (ECLS), mainly with extracorporeal membrane oxygenation (ECMO) [3]. The rewarming rate is the highest for ECMO with values up to 6 °C/h [4]. Due to limited availability and risk of severe complications, the use of ECMO is reserved for patients undergoing cardiopulmonary resuscitation or in deep hypothermia with signs of circulatory instability in select institutions [5]. On the contrary, RRT techniques are widely available and associated with a lower complication rate than ECMO therapy. However, no conclusive evidence on the efficacy of RRT in deep accidental hypothermia patients is currently available. This systematic review aims to assess the rewarming capacity of RRT in patients in severe hypothermia, below or equal to 32 °C.

## 2. Materials and Methods

This systematic review was registered in the PROSPERO International Prospective Register of Systematic Reviews (identifier CRD42021232821). A professional library assistant searched Embase, Medline, and Cochrane databases using the following keywords: “hypothermia” AND (“renal replacement therapy” OR “hemodialysis” OR “hemofiltration” OR “hemodiafiltration” OR “HD” OR “CVVH” OR “CVVHD” OR “CVVHDF”). The search included only articles in English with no time limit, up until 30 June 2021. The obtained results were independently assessed for their relevance by two authors (TD and PPod) according to the predefined criteria: only articles including human subjects with accidental moderate-to-severe hypothermia (defined as a body temperature below or equal to 32 °C) treated with RRT were included in the review. Reviews, animal studies, publications on therapeutic hypothermia or iatrogenic hypothermia were excluded. Conference abstracts were not analyzed. In addition, the references of the included studies were searched manually to find any additional studies. Any disagreements were resolved by team discussion. Two review authors (KM and SK) independently extracted data for each included study. The main objective was to summarize the RRT rewarming rates reported in the literature. Details about the type of RRT, other types of rewarming used, rewarming time, initial and end temperatures, the incidence of cardiac arrest, and patient outcome were also recorded. The quality of the publications included in the systematic review was assessed using the tool introduced by Murad et al. and presented in Table 1 [6].

For statistical analysis, we used StatsDirect software (StatsDirect Ltd., Merseyside, UK). Variable distributions were evaluated using the Shapiro–Wilk tests and quantile charts (Q–Q plot). To compare rewarming rates of different RRT techniques, we used the unpaired *t*-test. The results are presented as mean and 95% CI or median and IQR. For descriptive statistics, we used percentage and absolute value. Two-sided *p*-values < 0.05 were considered statistically significant.

## 3. Results

**Figure 1:**
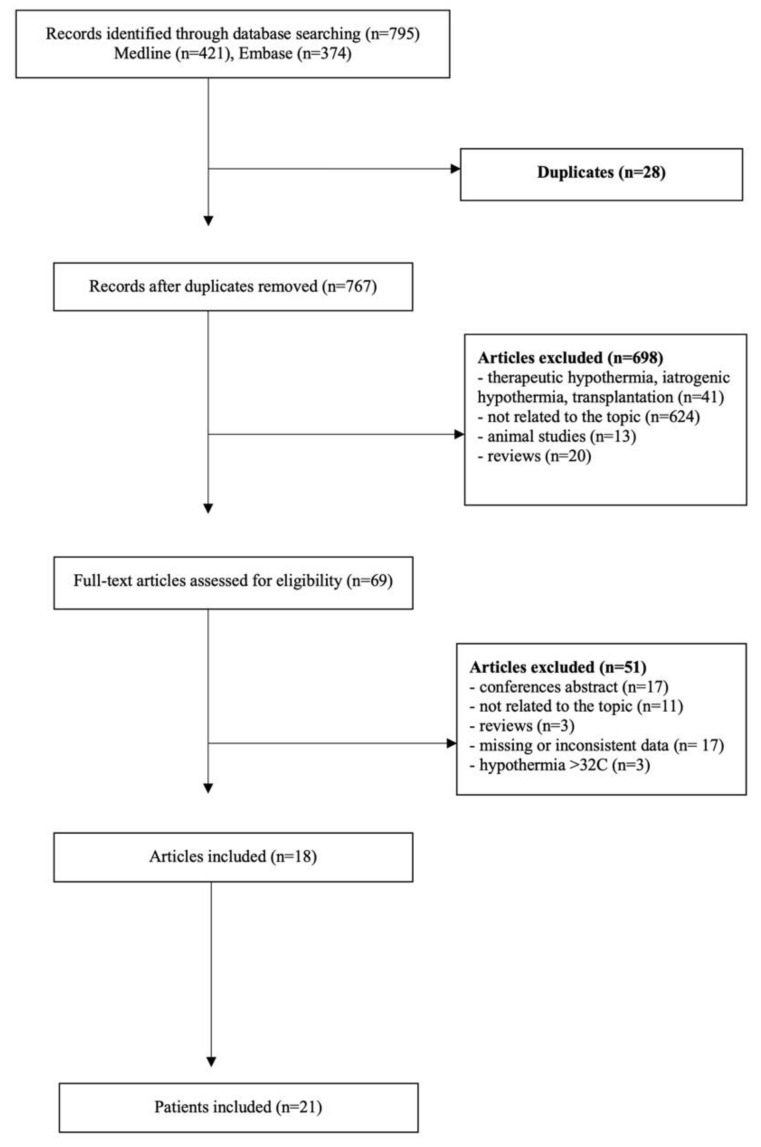
PRISMA flowchart.

From the 795 revised articles, 18 studies including 21 patients were selected for the final assessment and data extraction (Figure 1). The mean rate of rewarming calculated for all studies combined was 1.9 °C/h (95% CI 1.5–2.3) and did not differ between continuous (2.0 °C/h; 95% CI 0.9–3.0) and intermittent (1.9 °C/h; 95% CI 1.5–2.3) methods (*p* > 0.9). The lowest reported rewarming rate was 0.6 °C/h and the highest 4 °C/h, both were achieved with the continuous RRT [17,18]. For continuous renal replacement therapy (CRRT), the median rewarming time was 4.5 h (IQR 3.3–6.1), the initial median temperature 26.0 °C (IQR 22.5–30.4), and the final temperature 33.0 °C (IQR 30.6–35.5). The median rewarming time with intermittent renal replacement therapies (IRRT) was 3 h (IQR 2.7–4), and the temperature ranged 28.2 °C (IQR 27.3–28.8) to 33 °C (32.4–35).

The mean age of the patients was 49.5 years ranging from 17 to 77 years. Most patients were males (67%; 14/21) who were found outdoors (68%; 13/19), including one trauma patient. In two cases, the circumstances of hypothermia were not reported. Data on comorbidities were provided for less than half of the patients (43%; 9/21 patients) with alcohol abuse disorders predominating. The most frequently used RRT technique was hemodialysis (57%; 12/21), with dialysate temperature 37 °C (IQR 35–39 °C), median blood flow 250 mL/min (IQR 150–300 mL/min), and dialysate flow 500 mL/min (only in one publication dialysate flow was 800 mL/min [21]). Only two publications provided technical information on flows during CRRT, with a median blood flow of 175 mL/min (IQR 125–250 mL/min). Detailed information is presented in Table 2. 

Half of the reports did not specify the type of anticoagulation used, while in the remaining studies, heparin anticoagulation predominated (78%; 7/9). Four case reports included the information on coagulation laboratory tests (19%; 4/21), six (29%; 6/21) reported lactate levels with a mean value of 10.6 mmol/L (95% CI 2.6–18.6), nine (43%; 9/21) reported creatinine levels with a mean value of 1.0 mg/dL (IQR 0.7–2.5). The pH values were given variously (corrected and uncorrected for temperature) with a mean value of 7.15 (95% CI 7.03–7.28). Potassium concentration was reported in over half of the subjects (57%; 12/21) and was 3.8 mmol/L (95% CI 3.1–4.5). Nine patients (9/21; 43%) experienced cardiac arrest (CA) at different stages of the rescue procedures, with a survival rate of 55% (5/9) and good neurological outcomes in survivors. In the subgroup of patients with CA, continuous rewarming techniques predominated (56%; 5/9). Detailed data are available in Table 3.

## 4. Discussion

To our best knowledge, this systematic review is the first to provide structured evidence on the rewarming rates using RRT in hypothermic patients. The commonly cited rewarming rate during RRT is 2–3 °C/h [3]. Based on our review, the mean rewarming rate calculated for all studies combined was 1.9 °C/h and did not differ between continuous and intermittent methods. Interestingly, both the lowest rewarming rate and the highest rewarming rate were reported under continuous RRT techniques. Our study shows that renal replacement therapy may be an appealing alternative for rewarming of hypothermic patients. 

The first element in the chain of survival in patients suffering from accidental hypothermia is pre-hospital protection from cold, wet, and wind. The lack of adequate thermal insulation during transport poses the risk of further heat loss, thus may increase the risk of cardiac arrest (CA) [3]. The subsequent management of the hypothermic patient should be multidirectional and determined by factors such as the patient’s hemodynamic stability, the degree of hypothermia, and existing comorbidities [5]. There are no definite recommendations based on randomized trials regarding the patients’ eligibility for specific rewarming methods in the current literature. The current ERC/AHA resuscitation guidelines leave the decision for choosing the rewarming method to the physician, except for the cardiac arrest situation or hemodynamically unstable patients, in whom ECLS rewarming should be the method of choice [5,25]. Other therapies remain an option, not a recommendation, and each can be implemented not as the only main, but as an additional rewarming method. 

RRT is a promising form of extracorporeal rewarming, but there is little scientific evidence to support its use in this setting. The rewarming rate for active internal rewarming techniques varies, ranging from 1 °C/h for bladder lavage to over 6 °C/h for ECMO. These are mostly invasive techniques, but gaining access to the central compartment allows rapid rewarming and thus can minimize the risk of CA or, if it occurs, hasten the return of spontaneous circulation [3]. Every technique used should have the potential for rapid rewarming and minimal risk of inducing hemodynamic instability. For example, during a resuscitation attempt, pleural lavage may compromise the quality of chest compressions, which may not be desirable. It is also a common belief that extracorporeal devices which do not support the circulation (such as hemodialysis) are relatively contraindicated as they can compromise the hemodynamics and are ineffective in the absence of spontaneous circulation. However, as shown in this review, reports exist of RRT being used to rewarm a patient in CA with good neurological outcomes [16,18,19,22,23].

The ECMO therapy is the most effective in rewarming the hypothermic patient, both with preserved circulation and in CA. The device’s capabilities are greater than RRT. However, the optimal rewarming rates are still unknown. In patients in accidental hypothermia, mortality increases for every 0.5 °C/h drop in rewarming rate below 2 °C/h, but higher values cause a significant increase of neuronal damage markers and thus indicate the possibility of central nervous system injury [26,27]. Moreover, the use of ECMO poses the risk of severe and potentially fatal complications, both associated with severe bleeding and thromboembolism, whose incidence reaches almost 30% [28]. Therefore, ECMO therapy is reserved for highly specialized centers, often with cardiac surgery departments. 

Renal replacement therapy is a method of active internal rewarming, particularly interesting due to its widespread availability and relatively high rewarming potential. In addition to a much faster initiation time than ECMO [29,30], it is available not only in ICU’s but also in many other hospital units. Although the use of hemodialysis (HD) as a rewarming method was first described in 1965, only single cases have been reported in the literature since then [31]. As we have demonstrated, this surprisingly small number of publications provide limited details on the indications, duration, and specifics of the RRT protocols used in hypothermic patients.

The advantages of the RRT may be the relative simplicity and short onset time of the therapy, the ability of electrolyte correction, and precise control of fluid balance [32]. Also, using a smaller vascular cannula and avoiding systemic anticoagulation is beneficial. The use of systemic anticoagulation may hinder invasive procedures, such as pleural and peritoneal lavage, and may be contraindicated in trauma patients. Also, heparin anticoagulation should be avoided in hypothermic patients who have coagulopathy. For RRT, local anticoagulation with citrate is now the most commonly used method instead of systemic anticoagulation. It is the method of choice in critically ill patients, offering a relatively low (although not zero) risk of thromboembolic and hemorrhagic complications [33].

Currently, there is no uniform protocol for rewarming patients in hypothermia with renal replacement therapy. A recent study published by our team evaluated the heating capabilities of a CRRT device. We rewarmed a 5 L tank of 25 °C fluid (central compartment) using the CVVHDF technique. Initially, we assumed that the filter in which the dialysis fluid flows in the opposite direction to the blood might act as a countercurrent heat exchanger and be the potential element where the flowing blood can be rewarmed. However, according to mathematical calculations and acquired experimental data, we discovered that during CVVHDF, the filter is an inefficient heat exchanger and acts rather as a buffer—the blood flow (ml/min) was 60 times higher than the dialysate flow (ml/h). The main heat flux takes place on the uninsulated drains. We concluded that using two heaters instead of the single manufacturer’s heater (mounted on the inflow and outflow blood drains) allows the most efficient rewarming of the central compartment. 

We also found that substitution fluid at room temperature, which is used to replace ultrafiltrate volume, significantly reduces the blood temperature. Our study showed that the measured rewarming rate increases with fast blood flow and minimal replacement fluid flow. The median rewarming rate was highest during blood flow 150 mL/min and substitution 0 ml/h, reaching the maximum value of 6.0 °C/h (IQR 4.8–6.0) [34]. 

Worthy of note, the use of renal replacement therapy may carry a risk of hemodynamic instability, water-electrolyte, and acid-base disturbances. Also, even small size vascular access itself poses a risk of venous damage, hematoma, thrombosis, and hemorrhage. Moreover, in normothermic patients, the most frequently described adverse event of CRRT is hypothermia, occurring in up to 40% of the therapies [35]. Nowadays, RRT devices use various heating systems, including heating coils, heating sleeves, and heating systems incorporated directly into the bloodstream. This results in improved thermal efficiency of the devices and minimizes patient heat loss.

There are some limitations of our study. Due to the inclusion in the review of only case reports and case series, the risk of bias was rated high. Meta-analysis cannot be performed, and the result is very likely to be subject to publication bias. In addition, no attempt was made to contact the authors in the case of missing data. It should be emphasized that in the reviewed literature, RRT was never the only treatment provided, and the technical aspects and the RRT setting differed between the papers. Moreover, it is challenging to compare continuous methods such as CVVHDF with hemodialysis therapy. Flow rates of warm dialysate in HD ranged from 500 to 800 mL per minute, whereas flow rates in constant RRT are typically set in units of ml per hour. Therefore, they cannot be extrapolated to each other. 

## 5. Conclusions

The cases reported in the literature did not have sufficient detail to allow other researchers to replicate the study or to allow practitioners to draw conclusions related to their own practice. Based on the reviewed literature, it is currently not possible to provide high-quality recommendations for RRT use in specific groups of patients in accidental hypothermia. The calculated rewarming rate of 1.9 °C/h is the mean value for various RRT techniques, both continuous and intermittent. However, in analyzed studies, RRT was never the only rewarming technique for patients. While RRT appears to be a viable rewarming strategy, the choice of rewarming method should always be determined by the specific clinical circumstances, the available resources, and the current resuscitation guidelines. 

## Figures and Tables

**Table 1:** Quality assessment of included case reports and case series.

Authors/Year of the Publications	Selection	Ascertainment		Causality				Reporting
	1	2	3	4	5	6	7	8
Caluwe et al. 2010 [7]	●	●	●	n/a	n/a	n/a	●	●
Chen et al. 2005 [8]	●	●	●	n/a	n/a	n/a	●	●
Brodersen et al. 1996 [9]	●	●	●	n/a	n/a	n/a	●	●
Owda et al. 2001 [10]	●	●	●	n/a	n/a	n/a	●	●
Komatsu et al. 2007 [11]	●	●	●	n/a	n/a	n/a	●	●
Rahman et al. 2012 [12]	●	●	●	n/a	n/a	n/a	●	●
Hernandez et al. 1993 [13]	●	●	●	n/a	n/a	n/a	●	●
Sultan et al. 2009 [14]	●	●	●	n/a	n/a	n/a	●	●
Murakami et al. 2019 [15]	●	●	●	n/a	n/a	n/a	●	●
Hughes et al. 2007 [16]	●	●	●	n/a	n/a	n/a	●	●
Spooner et al. 2000 [17]	●	●	●	n/a	n/a	n/a	●	●
Puzio et al. 2020 [18]	●	●	●	n/a	n/a	n/a	●	●
Alfonzo et al. 2009 [19]	●	●	●	n/a	n/a	n/a	●	●
Carr et al. 1988 [20]	●	●	●	n/a	n/a	n/a	●	●
Singh et al. 2014 [21]	●	●	●	n/a	n/a	n/a	●	●
Wagner et al. 2008 [22]	●	●	●	n/a	n/a	n/a	●	●
Hagiwara et al. 2011 [23]	●	●	●	n/a	n/a	n/a	●	●
Van der Maten et al. 1996 [24]	●	●	●	n/a	n/a	n/a	●	●

1. Does the patient(s) represent(s) the whole experience of the investigator (center), or is the selection method unclear to the extent that other patients with a similar presentation may not have been reported? 2. Was the exposure adequately ascertained? 3. Was the outcome adequately ascertained? 4. Were other alternative causes that may explain the observation ruled out? 5. Was there a challenge/rechallenge phenomenon? 6. Was there a dose–response effect? 7. Was follow-up long enough for outcomes to occur? 8. Is the case(s) described with sufficient details to allow other investigators to replicate the research or to allow practitioners make inferences related to their own practice? ●—yes; ●—no; ●—not specified; n/a—not applicable.

**Table 2:** Review of hypothermia management with RRT in published case reports.

Authors/Year of the Publications	RRT	Anticoagulation	T_initial_ °C	T_target_ °C	Rewarming Rate °C/h	Initial/Ongoing Rewarming Technique	Technical Aspects	CPR	Survived
Caluwe et al. 2010 [7]	HD	No	28.8	35	1.1	wIVF	Qd (39 °C) 500 ml/min Q_b_ 300 ml/min	No	Yes
Chen et al. 2005 [8]	HD	NS	30.4	36.8	1.6	wB, wAir, wIVF, RH	NS	No	Yes
Brodersen et al. 1996 [9]	HF	NS	24	32	1.3	ns	Heater (37 °C) Q_b_ 180 ml/min	No	Yes
Owda et al. 2001 [10]	HD	No	30.2	36.7	1.9	wB, wIVF, PD	NS	No	Yes
Komatsu et al. 2007 [11]	CVVHDF	NS	26.8	33.0	1.4	wIVF, wAir	High room temperature Hot dialysate Shorten drains	No	Yes
Rahman et al. 2012 [12]	HD	NS	27.3	36.3	3.3	wB, wIVF, wAir	Q_d_ (37 °C) 500 ml/min Q_b_ 300 ml/min	No	Yes
Hernandez et al. 1993 [13]	HD	H	27.4	33	2.2	wIVF	Q_d_ (40 °C) 500 ml/min Q_b_ 450–500 ml/min	No	Yes
Sultan et al. 2009 [14]	HD	H	30.6 34.1*	33 36.3	2.1 1.1	wB, wIVF, WAir, BL	Q_d_ (35–36 °C) 500 ml/min Q_b_ 200 ml/min	No	Yes
Murakami et al. 2019 [15]	HD	N	28.3	32.4	1.6	wB, wIV	Q_d_ (35 °C) 500 ml/min Q_b_ 100–150 ml/min	No	Yes
	HD	H	28.3	32.7	1.5	wB, wIVF	Q_d_ (37 °C) 500 ml/min Q_b_ 150 ml/min	No	Yes
	HD	N	28.2	32	1.4	wB, wIVF	Q_d_ (35 °C) 500 ml/min Q_b_ 120 ml/min	No	No
Hughes et al. 2007 [16]	CVVH	NS	18	31	3.3	wIVF, wAir, BL G	blood warmed on outlet drain	Yes	Yes
Spooner et al. 2000 [17]	CVVH	H	30	34	4	wB, wIVF, GL, BL	NS	Yes	No
Puzio et al. 2020 [18]	CVVHD	NS	30.7	37.2	0.6	wIVF, wB, WE, BL, GL	NS	Yes	Yes
Alfonzo et al. 2009 [19]	CVVH	H	25.1	30.2	0.7	wB, wIVF, BL, GL, PL	blood drain heater (38.5 °C) Q_b_ 150–200 ml/min	Yes	Yes
Carr et al. 1988 [20]	HD	H	23.9	32.4	2.8	wB, wIVF, wAir	NS	Yes	No
Singh et al. 2014 [21]	HD	No	28	34	1.5	wB, wIVF, wAir, PlL, PL	Q_d_ (36–38 °C) 800 ml/min Q_b_ 400 ml/min	Yes	No
Wagner et al. 2008 [22]	CVVHD	NS	32	37	1.9	wIVF/other (NS)	Q_b_ 200–300 ml/min	Yes	Yes
Hagiwara et al. 2011 [23]	HD	NS	20	31.8	2.7	wB, wIVF	ECMO with no heater	Yes	Yes
Van der Maten et al. 1996 [24]	CVVHD	H	24	30	1.3	wB, wIVF, wAir, RH	Q_b_ 100–150 ml/min	Yes	No
	CVVHD	NS	21	33	2.4	wB, wIVF	NS	No	Yes

**T_initial_**—temperature of initiation RRT; **T_target_**—temperature of ending RRT; **CPR**—cardiopulmonary resuscitation, **RRT**—renal replacement therapy type; **CRRT**—continous renal replacement therapy; **HD**—hemodialysis; **HF**—hemofiltration; **CVVH**—continuous venovenous hemofiltration; **CVVHD**—continuous venovenous hemodialysis; **CVVHDF**—continuous venovenous hemodiafiltration; **N**—nafamostat mesilate; **H**—unfractioned heparin; **Q_d_**—dialysate flow rate; **Q_b—_** Blood flow rate; **wB**—warm blankets; **wIVF**—warm iv fluids; **wAir**—warm oxygen inspired; **RH**—radiant heat; **WE**—warm environment; **PL**—peritoneal lavage; **PlL**—pleural lavage; **PD**—peritoneal dialysis; **BL**—bladder lavage; **GL**—gastric lavage; **NS**—not specified; * HD was stopped between 33.1–34 °C due to clotting (rewarming rate in this temperature range was not included in the calculations).

**Table 3:** Patient characteristics.

Authors	Gender	Age	Situational Circumstances	Comorbidities	Neurological Status	BP (mmHg)	ECG	pH	pO2	pCO2	Glc	Coagulation	Cr	K	Lac	Hospital Stay (Days)	Neurological Outcome
Caluwe et al. 2010 [7]	M	71	Indoor	DM2 CHF CKD	GCS 9	NS	AV3 30 /min	7.09 uc	46.7	78.9	39	PT 61 %	2.5	5.8	NS	30	Good
Chen et al. 2005 [8]	M	60	NS	DM2 CHF HT AUD	U	117/52	NS 91 /min	7.32 ns	60.7	30.0	302	NS	7.3	4.6	NS	NS	Good
Brodersen et al. 1996 [9]	F	63	Indoor	NS	U	NS	STE 50 /min	7.61 c	NS	NS	219	NS	NS	2.9	NS	NS	Good
Owda et al. 2001 [10]	M	73	Outdoor	CHF CKD HT	U	NS	NS 25 /min	NS	NS	NS	NS	NS	NS	NS	NS	NS	NS
Komatsu et al. 2007 [11]	M	48	Outdoor	NS	GCS 6	NS	LQT J-waves	7.18 ns	NS	NS	NS	NS	NS	NS	13.4	6	Good
Rahman et al. 2012 [12]	M	45	Outdoor	AUD	D	110/73	Sinus J-waves 50 /min	7.27 ns	150	62	NS	NS	0.4	3.2	NS	5	Good
Hernandez et al. 1993 [13]	M	43	Outdoor	NS	U	50/30	Sinus 40 /min	7.11 c	45	19	NS	APT 81 s	2.4	3.9	NS	NS	Good
Sultan et al. 2009 [14]	M	65	Outdoor	NS	GCS 12	116/77	Sinus J-waves wQRS, ST changes 70 /min	7.27 ns	NS	NS	NS	NS	0.7	2.4	NS	2	Good
Murakami et al. 2019 [15]	M	48	Outdoor	AUD	GCS 8	114/99	Sinus J-waves LQT 48 /min	7.20 ns	NS	NS	60	NS	0.4	3.6	4.7	4	Good
	M	78	Indoor	NS	GCS 3	78/40	Sinus J-waves 39 /min	NS	NS	NS	54	NS	NS	NS	NS	NS	NS
	F	77	Indoor	NS	GCS 3	133/83	J-waves 63 /min	7.33 ns	NS	NS	NS	NS	NS	2.8	8.5	NS	Death
Hughes et al. 2007 [16]	F	17	Outdoor	NS	CPR	CPR	Asystole	7.17 uc	30.8	63.8	112	INR 2.4 APT ratio 1.6	NS	3.2	NS	11	Good
Spooner et al. 2000 [17]	F	77	Indoor	NS	GCS 8	93/55 CPR	Sinus 89 /min VF	NS	NS	NS	NS	NS	NS	NS	NS	1	Death
Puzio et al. 2020 [18]	F	25	NS	DM1 GD	CPR	CPR	NS	NS	NS	NS	NS	NS	NS	NS	NS	20	Good
Alfonzo et al. 2009 [19]	F	23	Outdoor	NS	GCS 3	CPR	PEA VF	NS	NS	NS	NS	NS	1*	NS	NS	16	Good
Carr et al. 1988 [20]	M	35	Outdoor	Quadri-plegia	CPR	CPR	VF	7.24 c	NS	43.8	NS	PT 11.7 s PTT 49 s	NS	NS	NS	1	Death
Singh et al. 2014 [21]	M	49	Indoor	AUD	CPR/ROSC	160/90	NS 58 /min	6.80 ns	90	38	NS	NS	2.5	4.4	10.6	1	Death
Wagner et al. 2008 [22]	M	39	Outdoor (trauma)	NS	GCS 12	67/37CPR	NS 100 /min	6.91 ns	NS	NS	NS	NS	NS	NS	NS	54	Good
Hagiwara et al. 2011 [23]	F	30	Outdoor	NS	CPR	CPR	VF	NS	NS	NS	NS	NS	NS	NS	NS	NS	Good
Van der Maten et al. 1996 [24]	M	46	Outdoor	None	CPR	CPR	VF	6.72 ns	NS	NS	149	NS	1	5.4	24	5.5 h	Death
	M	27	Outdoor	NS	GCS 3	80/45	Bradycardia	7.06 ns	350	78	423	NS	NS	3.5	2.6	3	Good

**BP**—blood pressure; **pCO2**—partial pressure of carbon dioxide (mmHg); **pO2**—partial pressure of oxygen (mmHg); **Glc**—serum glucose (mg/dL); **Cr**—serum creatinine (mg/dL); **K**—serum potassium (mmol/L); **Lac**—serum lactates (mmol/L); **CHF**—chronic heart failure; **CKD**—chronic kidney disease; **AUD**—alcohol use disorders; **GD**—Graves’ disease; **HT**—hypertension; **DM1**—diabetes mellitus type 1; **DM2**—diabetes mellitus type 2; **A**—alert; **U**—unconscious; **D**—disorientated; **GCS**—Glasgow Coma Scale; **AV3**—third degree atrioventricular block; **LQT**—long QT; **STE**—ST segment elevation; **wQRS**—wide QRS complex; **CPR**—cardiopulmonary resuscitation; **ROSC**—return of spontaneous circulation; **VF**—ventricular fibrillation; **pH “uc”**—pH uncorrected for temperature; **pH “c”**—pH corrected for temperature; **“NS”**—not specified; * approximate value.

## Data Availability

Data is contained within the article.
